# Frequency and Pathological Phenotype of Bovine Astrovirus CH13/NeuroS1 Infection in Neurologically-Diseased Cattle: Towards Assessment of Causality

**DOI:** 10.3390/v9010012

**Published:** 2017-01-18

**Authors:** Senija Selimovic-Hamza, Céline L. Boujon, Monika Hilbe, Anna Oevermann, Torsten Seuberlich

**Affiliations:** 1 Graduate School for Cellular and Biomedical Sciences, University of Bern, Bern CH-3012, Switzerland; senija.selimovic@vetsuisse.unibe.ch; 2 DCR-VPH, Division of Neurological Sciences, NeuroCenter, University of Bern, Bern CH-3012, Switzerland; celine.boujon@vetsuisse.unibe.ch (C.L.B.); anna.oevermann@vetsuisse.unibe.ch (A.O.); 3 Institute of Veterinary Pathology, Vetsuisse-Faculty, University Zurich, Zurich CH-8057, Switzerland; hilbe@vetpath.uzh.ch

**Keywords:** astrovirus, bovine, encephalitis, neurotropic, infection, cattle

## Abstract

Next-generation sequencing (NGS) has opened up the possibility of detecting new viruses in unresolved diseases. Recently, astrovirus brain infections have been identified in neurologically diseased humans and animals by NGS, among them bovine astrovirus (BoAstV) CH13/NeuroS1, which has been found in brain tissues of cattle with non-suppurative encephalitis. Only a few studies are available on neurotropic astroviruses and a causal relationship between BoAstV CH13/NeuroS1 infections and neurological disease has been postulated, but remains unproven. Aiming at making a step forward towards assessing the causality, we collected brain samples of 97 cases of cattle diagnosed with unresolved non-suppurative encephalitis, and analyzed them by in situ hybridization and immunohistochemistry, to determine the frequency and neuropathological distribution of the BoAstV CH13/NeuroS1 and its topographical correlation to the pathology. We detected BoAstV CH13/NeuroS1 RNA or proteins in neurons throughout all parts of the central nervous system (CNS) in 34% of all cases, but none were detected in cattle of the control group. In general, brain lesions had a high correlation with the presence of the virus. These findings show that a substantial proportion of cattle with non-suppurative encephalitis are infected with BoAstV CH13/NeuroS1 and further substantiate the causal relationship between neurological disease and astrovirus infections.

## 1. Introduction

The first interest in viral infections of the central nervous system (CNS) dates back to antiquity [1], but neurovirology has only recently become a growing field with the discovery of less invasive techniques of surgery and diagnostic sampling in the late 20th century. Since the invention of new molecular tools and methods, like PCR and next generation sequencing (NGS), the spectrum of potential infectious agents has expanded, both in animals and in humans, providing new directions in investigating etiologically unresolved cases or diseases.

Astroviruses are small, non-enveloped, single-stranded RNA viruses with a star-like shape when viewed in the electron microscope. Since the discovery of the first astrovirus in a human stool sample in 1975 [2], astroviruses have been described in several mammalian and avian species and are known as a cause of diarrhea and gastroenteritis in children [3]. Recently, growing evidence of neurotropic astrovirus species has arisen in cases of encephalitis and meningitis in humans and animals [4,5,6,7,8,9,10,11]. Non-suppurative encephalitis is an inflammatory pattern observed in the context of post-mortem histopathological brain examinations and is indicative of viral infections. It is characterized by perivascular cuffs composed of mononuclear cells, by gliosis, and by neuronal necrosis. In Switzerland, non-suppurative encephalitis has been diagnosed in approximately 15% of neurologically-diseased cattle, but in many cases the etiology could not be determined and remained unknown [12,13,14,15]. These cases occurred sporadically and in single animals and were designated as “European sporadic bovine encephalitis” (ESBE). However, it has yet to be determined whether these cases of non-suppurative encephalitis were indeed caused by a single virus, or instead by several different viruses.

Using NGS, BoAstV has been found in the brains of cattle with non-suppurative encephalitis, termed BoAstV CH13 or BoAstV NeuroS1 [6,7]. As both virus isolates represent the same genotype species, it is hereafter referred to BoAstV Ch13/NeuroS1 [16]. In small-scale retrospective studies, BoAstV CH13/NeuroS1 was found in the brains of 5/22 and 3/32 cattle with unresolved non-suppurative encephalitis in Switzerland and in the USA, respectively. These results have provided a first hint towards the possible association of the virus with the disease, but did not allow for robust conclusions.

**Figure 1:**
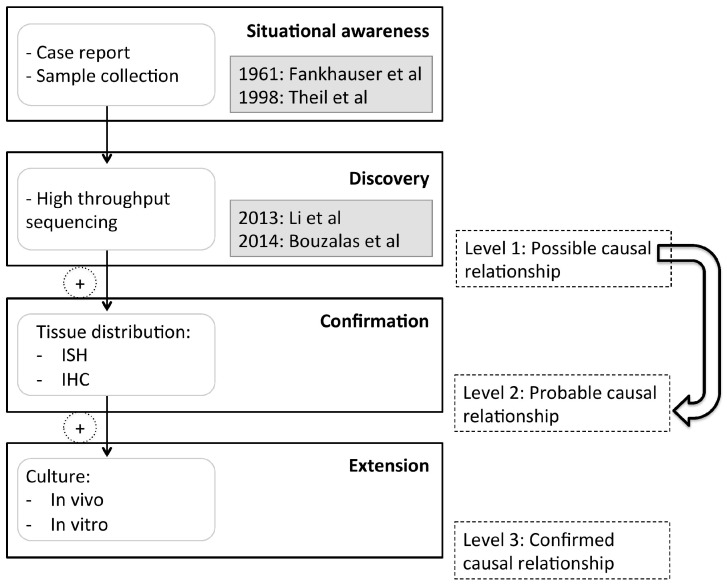
Assessment of causation of bovine astrovirus (BoAstV) CH13/NeuroS1 infection in non-suppurative bovine encephalitis (Figure adapted from: Lipkin and Anthony [18]). The first reports of non-suppurative encephalitis in cattle date back to 1961, but all studies aiming at identifying a possible agent have remained inconclusive [12,13,14]. After discovery of BoAstV CH13/NeuroS1 in such cases, the criteria for a “possible causal relationship” were met but not yet for a probable causal relationship due to limitations in the study design [6,7]. To confirm that the BoAstV CH13/NeuroS1 is a probable cause for bovine non-suppurative encephalitis, we investigated the statistical association of the virus and the disease, and compared the topography of virus infection to that of the histopathological brain lesions.

The postulates of Koch are still considered the gold standard to prove causal relationships of microbes and diseases. These postulates define the following requirements for proof of causation: (i) microorganisms are associated with a disease and its lesions, (ii) microorganisms can be isolated from the host and purely cultured, and (iii) the disease is reproducible by inoculation of healthy hosts and that the same microorganism can then be isolated from that host [17]. While these criteria remain an ideal to strive for, the conditions cannot always be met. Not all viruses readily replicate in cell culture systems and brain samples of diseased individuals are often not available. In consequence, many cases of viral infection of the CNS remain unresolved, leaving us largely unaware of potential threats for animal and public health. In 2015, Lipkin and Anthony suggested a refined classification for the assessment of causation, which is adapted to the situation in virus discovery by NGS [18]. Criteria are defined to determine (i) a possible causal relationship (level 1) or (ii) a probable causal relationship (level 2), even in the absence of a culture system for virus propagation, and they include the effect of vaccines and specific drugs into the final level (level 3) of confirmed causation (Figure 1). While a causal relationship is regarded as possible if a virus has been discovered in one or several diseased individuals, the relationship becomes probable with a statistical association of the virus with the disease, and when the virus is found at high concentration at the site of pathology.

For BoAstV CH13/NeuroS1, the postulates of Koch have not yet been fulfilled, because the virus cannot be isolated in cell culture and a reverse genetics system is not yet available. In the present study, we aimed at taking the first step towards assessing causality, by following the suggestions of Lipkin and Anthony. We performed a large-scale retrospective investigation of unresolved cases of non-suppurative encephalitis in order to assess the statistical association of BoAstV CH13/NeuroS1 infection and neurological disease and its neuroanatomical correlation to the pathological lesions in cattle. The results provide important insights on the frequency of BoAstV CH13/NeuroS1 infection in the Swiss cattle population and further support it being a major cause of encephalitis in cattle.

## 2. Materials and Methods

A total of 97 cases of histologically-confirmed bovine non-suppurative encephalitis of unresolved etiology were collected from the archives of the Vetsuisse Faculty in Bern and Zurich, Switzerland. All animals diagnosed were presenting with neurological symptoms, mainly gait abnormalities and behavioral changes. As negative controls, we analyzed samples of different brain regions of healthy animals without brain lesions (*n* = 52), and of animals with neurological disorders but with lesions indicating a bacterial origin (*n* = 6), under the same conditions. All cases were submitted to the neuropathological diagnostic services between 1985 and 2015. Tissue samples included formalin-fixed paraffin-embedded (FFPE) tissue of the CNS, mainly the brainstem, cerebellum, cerebrum and hippocampus. All tissues were analyzed by in situ hybridization (ISH) for the presence of BoAstV CH13/NeuroS1 RNA, and positive results were confirmed by immunohistochemistry (IHC). Tissues of cases with a known BoAstV CH13/NeuroS1 positive and negative status served as positive and negative controls.

A non-radioactive digoxygenin-labeled ISH was done according to the protocol of Bouzalas et al. [7], using two ISH-probes (A and B) that are complementary to the 5’ end of open-reading frame (ORF) 2 and the center of ORF2 of the BoAstV CH13/NeuroS1 genome. Briefly, after deparaffinization, rehydration and 0.2M hydrogen chloride (HCL) treatment, we added Proteinase K (Roche, Mannheim, Germany), deactivated it with 4% paraformaldehyde, and incubated the tissue sections in a prehybridization mix before the hybridization-step and the application of the ISH-probes A and B on serial sections overnight. Probe binding was detected by anti-digoxygenin-AP-Fab fragments (Roche) and nitro blue tetrazolium (NBT)/5-bromo-4-chloro-3-indolyl-phosphate (BCIP) substrate (Roche).

IHC was performed according to the validated ORF2-con protocol [19] and each run included negative and positive control sections. The hyperimmune serum ORF2-con detects viral epitopes on the conserved N-terminus of the capsid protein of BoAstV CH13/Neuro-S1. After deparaffinization, dehydration of the FFPE-tissue and peroxidase blocking in 3% H_2_O_2_ we used microwave heating (20 min at 95 °C) in Dako Target Retrieval solution, pH 9 (Agilent Technologies, Santa Clara, California, USA) as antigen retrieval method. Blocking was performed with 10% normal goat serum in phosphate-buffered saline (PBS)-Tween solution, after which the ORF2-con immunoserum (1:100) was added overnight at 4 °C. Antibody binding was detected by the Dako REAL Detection System according to the instructions of the manufacturer (Agilent Technologies). IHC and ISH grading was performed according to the number of stained neurons. Serial sections of all slides were submitted to hematoxylin-eosin staining for histopathological assessment of lesions.

ISH and IHC results were assessed using a semi-quantitative grading: (−) = no positive cells; (+) = 1–10 positive cells; (++) = 10–50 positive cells, and (+++) = >50 positive cells. Histopathological lesions were similarly graded as on haematoxylin and eosin (HE) stained slides: (−) = no lesions, (+) = mild lesions, (++) = moderate lesions, and (+++) = severe lesions. The assessment of both the ISH/ IHC and the histopathological grading was not performed on specific microscopic fields but on complete sections. The topographical correlation of lesions with BoAstV CH13/NeuroS1 detection was judged as good correlation, partial correlation, and the absence of correlation. A good correlation is seen in sections where the virus is located in the same microscopic field, affecting the same neuroanatomical area that shows the highest grade of lesions. A partial correlation does show an overlap in the presence of lesions and the virus, while not precisely the same, but a close-by neuroanatomical area is affected, or when major lesions comprise glial nodes or perivascular cuffs, but not neurons. Absence of correlation reflects a very large discrepancy between lesions and the presence of viral RNA (vRNA) or proteins [20].

Statistical analysis was done by Fisher’s exact test using the Prism software (Version 5; GraphPad, La Jolla, California, USA).

## 3. Results

### 3.1. Frequency of Bovine Astrovirus (BoAstV) CH13/NeuroS1 Infection

The ISH results of all available brain regions of 97 cases of bovine non-suppurative encephalitis revealed that 33 cases were positive for BoAstV CH13/NeuroS1 RNA (34%) by both ISH probes and in at least one region of the CNS, while none of the 58 negative controls showed reactivity, neither with ISH-probe A, nor with probe B, under the same conditions (Table 1). In BoAstV CH13/NeuroS1-positive cases, the vRNA was detected predominantly in the grey matter, in the form of a deep purple staining in the cytoplasm of neurons. Positive controls showed a comparable staining. Although not all brain regions of interest were available for all cases, it appears that the neuroanatomical distribution of the vRNA varies between individual animals and shows no clear phenotype, indicating that the virus might be present throughout the grey matter of the CNS with no preferred target region.

We analyzed all 33 cases that were BoAstV CH13/NeuroS1-positive in the ISH by IHC to confirm the results. For this we chose the sections with the strongest positive ISH signal from each case and compared the IHC result directly to the ISH result. In 27 BoAstV CH13/NeuroS1 positive cases the staining in the IHC was comparable to the one in the ISH, not only in the region but also in the number of cells affected by the virus. It appears that approximately the same number of neurons showed a labeling in the same neuroanatomical region by both methods. However, in six astrovirus-positive cases we could not detect an ORF2-con specific signal in the IHC despite a strong neuronal staining of a serial section by ISH. Based on repetitively positive signals by both ISH-probes in at least two brain regions, we still considered these cases as BoAstV CH13/NeuroS1-positive.

Taken together, the BoAstV CH13/NeuroS1 infection that we detected by ISH and IHC in approximately 1/3 of cattle diagnosed with non-suppurative encephalitis, while negative controls remained unstained, clearly shows a correlation with the disease, which is supported by an extremely highly significant statistical correlation (two-tailed *p* < 0.0001).

### 3.2. Anamnestic Clinical Data

**Figure 2:**
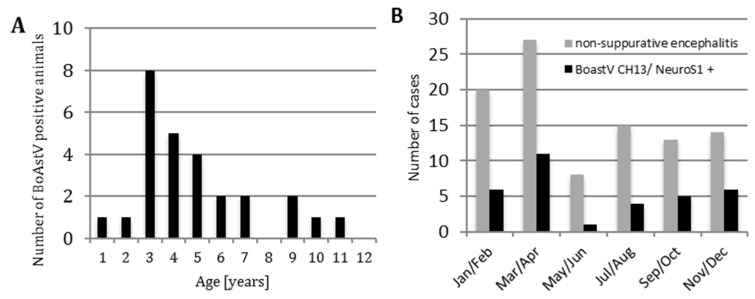
Age distribution (**A**) and seasonal distribution (**B**) of BoAstV CH13/NeuroS1 positive cases.

The clinical data of the collected cases was not complete to conduct a detailed analysis of clinical symptoms, but information on the age, sex, and date of death was available for most animals. According to the literature, ESBE has been diagnosed in cattle up to the age of nine years, but in a majority of cases, is usually observed at the age of two years [12]. The mean age of BoAstV CH13/NeuroS1-infected animals in our study was 3.8 years. The majority (63%) of virus-positive cattle were aged between two and four years (Figure 2A). The information on sex was available for 28 BoAstV CH13/NeuroS1 positive cases. Interestingly, 27 BoAstV CH13/NeuroS1 positive cases were female and only one case was a steer. In astrovirus-negative cases the distribution of sexes was similar (Supplementary Table S1).

The study of Theil et al. describes seasonal effects in the frequency of cases of non-suppurative encephalitis, which were mainly detected in winter and spring [12]. This was also the case for the total number of encephalitis cases in our study. The seasonal distribution of BoAstV CH13/NeuroS1-positive cases showed a similar distribution with a continuously growing number of astrovirus-positive animals from the beginning of winter until the end of spring. The percentage of BoAstV CH13/NeuroS1-positive cases among the reported cases of non-suppurative encephalitis peaked in November/ December and March/ April (Figure 2B).

In a total of 32 cases with non-suppurative encephalitis, clinical anamnestic data was available, among which eight cases had a BoAstV CH13/NeuroS1-positive status. The duration from the onset of symptoms until death or euthanasia was variable, both in astrovirus-positive and -negative cases, ranging from one day up to several weeks. Typical symptoms included gait abnormalities, behavioural changes, fever, salivation, and gastrointestinal problems, but the specific clinical picture varied by case. However, when comparing the astrovirus-negative and the astrovirus-positive group, there were some differences in the symptomology. Astrovirus-positive animals had higher instances of behavioral changes (such as anxiety or aggression), pathologies of the gastrointestinal tract (including diarrhea or excessive salivation), and nystagmus. On the other hand, astrovirus-negative non-suppurative cases had slightly higher instances of gait abnormalities, opisthotonus, ataxia, fever, and recumbency (Supplementary Figure S1).

### 3.3. Neuroanatomical Distribution of BoAstV CH13/NeuroS1

**Figure 3:**
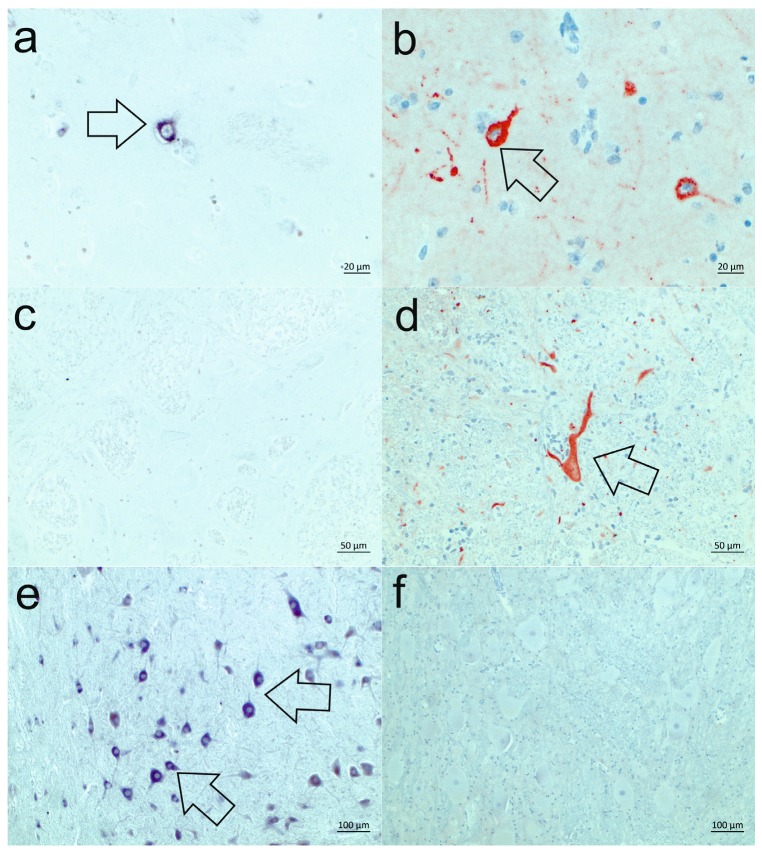
Comparison of the detection of the viral RNA (vRNA) by in situ hybridization (ISH) and of the viral proteins by immunohistochemistry (IHC). The vast majority of analyzed sections show a good correlation of ISH and IHC, with a comparable staining in neurons, as seen in the cerebrum of case 50898 (**a**, **b**; examples are indicated by arrows). Only in three sections can a lack of correlation be observed, with either a negative ISH-result (**c**), or a red, granular staining in the IHC (**d**, arrow) as seen in the brainstem of case 50898, or vice versa, in case 25018: a deep purple ISH staining in neurons of the brainstem (**e**, arrows) is observed, while the IHC remains negative (**f**).

The neuroanatomical distribution of the vRNA and proteins was evaluated in selected cases (*n* = 10) in which a whole set of representative brain regions was available by ISH and IHC on serial sections (Table 2). The majority of cases showed a similar BoAstV CH13/NeuroS1 positive signal with both methods. Only in two cases the results of the ISH and the IHC were divergent in at least one brain region (Figure 3). In case 25018 the IHC did not give a positive result at all (Figure 3f), whereas in case 50898 the IHC showed a strong reaction in the brainstem that was previously classified as ISH-negative (Figure 3d). However, since the majority of analyzed sections in this study showed a high degree of accordance in the detection of vRNA by ISH and viral proteins by IHC, these results show that both techniques indeed detect BoAstV CH13/NeuroS1-infected cells with high sensitivity and specificity.

**Figure 4:**
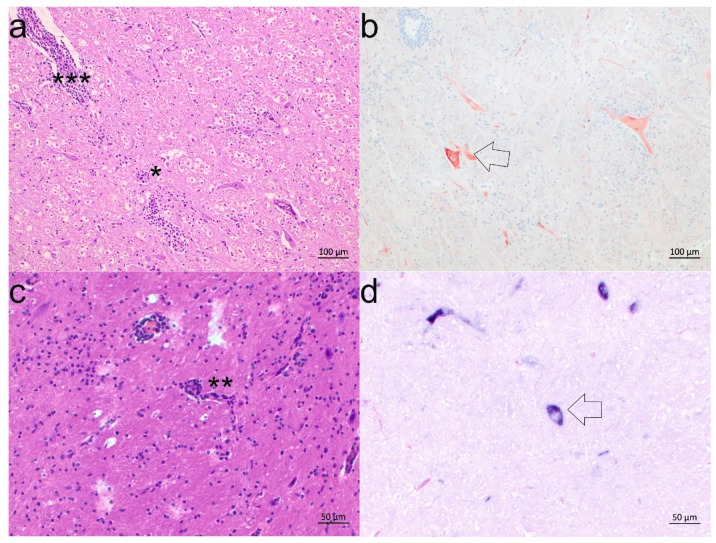
Correlation of pathological lesions and presence of the virus. Brainstem sections of two BoAstV CH13/NeuroS1-positive animals show glial nodes (*), neuronal necrosis (**), and perivascular cuffing (***) typical for non-suppurative bovine encephalitis in HE-stained sections. Severe lesions in the brainstem of case 50898 (**a**) show a good correlation to the results from the viral detection by IHC in most parts of the brainstem (**b**, arrow). In the brainstem of case 26875 perivascular cuffs are observed (**c**), correlating well to the BoAstV CH13/NeuroS1 positive staining of neurons in ISH in the same area (**d**, arrow).

To assess whether the requirements for a probable causal relationship are fulfilled, we then compared the presence of the virus with that of the major brain lesions. Interestingly, when taking a closer look at the comparison of ISH, IHC, and HE staining of serial sections of the ten selected cases, all cases show a good spatial correlation between virus detection and the presence of lesions in at least two different brain regions (Figure 4; Table 2). However, there were single sections in which the total of virus infected cells was high, but only mild pathological changes were observed, and vice versa, suggesting different stages of infection in these regions. In particular, the cases 24231, 24594, 24595, and 43660 showed moderate to severe pathological changes in combination with an absence or low levels of viral load presence in some brain regions, pointing towards the possibility of rapid viral clearance (Table 2).

Previous studies have already indicated a possible association of the BoAstV CH13/NeuroS1 and the histopathological lesions characteristic for non-suppurative encephalitis. However, these data were incomplete because they were only based on single brain tissue sections or cases without a systematic investigation of all available brain regions [6,7]. The present study further supports the probability of causality by showing a consistently high correlation of virus and lesions in a vast majority of brain regions among BoAstV CH13/NeuroS1 positive cases of non-suppurative bovine encephalitis.

### 3.4. Cell Tropism

Based on morphological criteria we concluded that the major targets for BoAstV CH13/NeuroS1 infection are neurons. Purkinje cells, which are a specific subset of neurons of the cerebellum, however, did not reveal a positive signal by ISH or IHC in the any of the cases under investigation. These findings are in contrast to the study by Li et al., in which there was evidence of the BoAstV CH13/NeuroS1 mainly affecting Purkinje cells in the cerebellum of the cattle with encephalitis [6]. In a previous study in an immunocompromised human, astrocytes were identified as the main target of a neurotropic astrovirus and in our previous studies, we were able to detect astrovirus-positive microglia in one case [5,20]. Based on cell morphology, there is no evidence of virus-infected astrocytes or microglia in BoAstV CH13/NeuroS1 positive cases in the present study. These results are important, because they indicate the type of cells that should be considered for establishing cell culture models for virus propagation, which is a prerequisite to further assess virus-disease causality.

## 4. Discussion

Using different methods of virus detection in FFPE brain tissues, we have performed a retrospective study on the frequency and neuroanatomical distribution of the neurotropic astrovirus BoAstV CH13/NeuroS1 in 97 cases of cattle diseased of non-suppurative encephalitis of unknown etiology and 58 negative controls, so as to further assess the causal relationship of virus infection and disease. The BoAstV was detectable in 34% of analyzed cases, but none in healthy animals, indicating a significant correlation to the disease. The histopathological findings in hematoxylin-eosin stained serial sections occur mainly in the very same areas where vRNA or proteins were detected by ISH and IHC, and in a majority of sections they are of a comparable intensity, which further substantiated the probable causal relation.

However, we observed exceptions in single sections of the brain where pathological lesions are present, but no virus was detectable, or vice versa. Since animals under investigation were at different stages of the disease and the intensity of lesions was variable between individuals, we propose that different CNS infection stages may be accountable for these results.

The BoAstV CH13/NeuroS1 was detectable in all parts of the CNS, but the intensity of infection was variable across cases. As it is known that bovine non-suppurative encephalitis shows a variation of intensity of perivascular cuffs, gliosis, and neuronal necrosis throughout the brain and brainstem [12], our findings by ISH and IHC were to be expected, particularly since the presence of the virus mostly correlates to the pathology. Still, we cannot define a clear neuropathological phenotype of infection and suggest testing different brain regions in suspect cases to not miss a BoAstV CH13/NeuroS1 positive case. Although the brainstem does seem like a favorable region for testing since the majority of cases reveal a BoAstV CH13/NeuroS1 positive signal in this region, there are exceptions that urge for additional tests of at least one more brain region, preferably the cortex or hippocampus.

In all cases in this study, the BoAstV CH13/NeuroS1 shows a cell tropism for neurons only. vRNA or proteins could not be detected in microglia or astrocytes in any of the investigated cases, indicating that these cells might not be the major target. This result is in line with one of our previous studies, where there is evidence of BoAstV CH13/NeuroS1-positive glial nodes only in one case [20].

It is known that enteric astrovirus infection in humans mainly occurs in winter [21], but our study provides evidence that neurotropic isolates might also cause more clinical infections from the onset of winter to the period of spring. Nevertheless, this result is to be interpreted with caution, because the number of cases of non-suppurative bovine encephalitis normally drops during summer months. It is important to note that cattle in Switzerland are usually kept in the Alps during the summer and neurological or behavioral changes might not be detected or reported in a timely manner by the farmers, which could have an impact on studying seasonal effects of virus infection. However, it is reasonable to conclude that affected cattle are mainly of a younger age (3–5 years), and preliminary results of another ongoing clinical study show that in most cases, astrovirus-infected cattle suffer from gait abnormalities and changes in behavior (data not shown). The full clinical picture is still unresolved and therefore we would strongly urge for an enhancement to the system of reporting neurologically-diseased cattle and for considering the BoAstV CH13/NeuroS1 infection already at the stage of primary veterinary consultations in the field.

Although a high proportion of cases of non-suppurative encephalitis show BoAstV CH13/NeuroS1 infection in the CNS, approximately two thirds of the cases still remain unresolved. In previous studies, we have identified other viruses in such cases, among them another astrovirus, BoAstV CH15, and a report from Germany reported a very similar virus in the brain of a diseased cow [9,10,22]. It is possible that more divergent, and unknown astroviruses could also cause viral encephalitis, but have gone undetected so far.

In conclusion, our results provide further evidence for a probable causal relationship between BoAstV CH13/NeuroS1 and neurological disease. The proof of causation is still needed and depends largely on successful virus isolation in cell culture and experimental induction of the disease. First attempts to propagate the virus in colorectal adenocarcinoma cells (Caco-2), which are frequently used for human enteric astroviruses [23], and in fetal bovine brain cells (FBBC-1) were inconclusive, but more comprehensive studies are under way in our laboratory. In addition to this, it is necessary to study the route of infection in diseased animals and include other organ systems, outside the CNS, in the analysis. Only in two of the BoAstV CH13/NeuroS1 positive cases did we have sections of lung, liver, and intestines available, but none of these samples was positive for vRNA or proteins. A systematical investigation of other organs in a significant number of BoAstV CH13/NeuroS1 positive cases would be necessary to give an insight into the broader pathology of this disease.

Studies aiming at understanding other possible viruses causing bovine non-suppurative encephalitis, at developing in vitro systems, as well as tools for serological diagnostics and research into disease epidemiology, should be encouraged to further expand our understanding of the causes and significance of neuroinfectious diseases, which potentially threaten animal and public health.

## Figures and Tables

**Table 1:** Frequency of the bovine astrovirus (BoAstV) CH13/NeuroS1 detection in cases of non-suppurative bovine encephalitis and a control group by in situ hybridization (ISH). The BoAstV CH13/NeuroS1 was detected only in diseased animals with a significant statistical correlation, but not in control animals previously diagnosed as healthy or as encephalitis of bacterial origin.

	BoAstV CH13/NeuroS1 ISH		Total
Non-suppurative encephalitis	+	-	
+	33	64	97
-	0	58	58
Total	33	122	155

**Table 2:** Detection of the BoAstV CH13/NeuroS1 by in situ hybridization (ISH) and immunohistochemistry (IHC), evaluation of severity of histopathological lesions, and topographical correlation of viral presence to histopathological lesions.

Case No.	Brainstem			Cerebellum			Cerebrum			Hippocampus		
	HE	ISH	IHC	HE	ISH	IHC	HE	ISH	IHC	HE	ISH	IHC
24231	+++	-	-	+++	+++	+++	-	+	+	+++	+++	+++
24594	++	++	++	+++	++	+	++	+++	++	++	++	+
24595	+++	+	+	+++	+	+	+++	++	++	+++	+++	++
25018	+	+++ *	- *	+++	++ *	- *	-	-	-	-	-	-
26730	++	+	++	+++	++	+	-	+	n.a.	++	+++	++
26875	++	++	+++	+++	+	+	+	++	+	+	+	+
42799	+++	++	++	+++	+	+	++	+	+	+++	-	-
43660	++	+	+	-	-	-	-	-	-	++	-	-
45664	+++	+	+	+++	+	+	+++	++	++	+++	++	++
50898	+++	- *	++ *	+++	++	++	+	+	++	+	++	++

*, Sections with no topographical correlation of ISH and IHC; n.a., not available. Criteria for grading as (−) absent, (+) weak, (++) moderate, and (+++) strong are defined in Section 2. Topographical correlation of ISH/IHC labeling with pathological lesions: good correlation, dark grey; partial correlation, light grey; white, no correlation.

## Supplementary Materials

The following are available online at www.mdpi.com/1999-4915/9/1/12/s1. Table S1: Descriptive statistics for 33 cases of non-suppurative encephalitis considered BoAstV CH13/NeuroS1-positive; Figure S1: Anamnestic clinical information on non-suppurative encephalitis cases and BoAstV Ch13/NeuroS1-positive cases.
