# Supplementary material for: Frequency and Pathological Phenotype of Bovine Astrovirus CH13/NeuroS1 Infection in Neurologically-Diseased Cattle: Towards Assessment of Causality
Source: Viruses. 2017 Jan 18;9(1):12. doi: 10.3390/v9010012 (PMC5294981; doi:10.3390/v9010012)
Supplement: Supplementary file 1 [file viruses-09-00012-s001.pdf]

Supplementary Materials: Frequency and Pathological Phenotype of Bovine Astrovirus CH13/NeuroS1 Infection in Neurologically-Diseased Cattle: Towards Assessment of Causality

Senija Selimovic-Hamza, Céline L. Boujon, Monika Hilbe, Anna Oevermann and Torsten Seuberlich

Table S1. Descriptive statistics for 33 cases of non-suppurative encephalitis considered BoAstV CH13/NeuroS1-positive. In 16 cases, frozen tissue of the brainstem was available for further testing by RT-PCR. The RT-PCR results matching the results from the in situ hybridization (ISH) and immunohistochemistry (IHC) in all cases, except in case no. 25018 (ISH-positive, IHC-negative, RT-PCR-negative) and 50898 (ISH-positive, IHC-negative, RT-PCR-positive). In nine cases, data from next-generation sequencing (NGS) confirmed the presence of the BoAstV CH13/NeuroS1 in previous studies.

Case Number	BoAstV CH13/NeuroS1	Date of Diagnosis	Age in Years	Sex	Frozen Material	NGS Data	RT PCR
20030	+	04.04.89	1.5	n.a.	-	-	-
21802	+	18.12.91	n.a.	f	-	-	-
22770	+	01.06.93	6	f	-	-	-
23120	+	23.11.93	9	f	-	-	-
23675	+	17.10.94	n.a.	m	-	-	-
23871	+	20.02.95	2	f	a.	a.[1]	positive[3]
23985	+	25.04.95	2.5	f	a.	a.[1]	positive[3]
24203	+	28.08.95	n.a.	f	-	-	-
24231	+	18.09.95	0.25	n.a.	-	-	-
24250	+	03.10.95	5	f	a.	-	negative[3]
24586	+	18.03.96	4	f	a.	-	negative[3]
24594	+	23.03.96	10	n.a.	-	-	-
24595	+	23.03.96	3.5	f	-	-	-
24903	+	16.08.96	3	n.a.	-	-	-
25018	+	07.11.96	8	n.a.	a.	-	negative[2]
26730	+	27.02.98	4	f	a.	a.[1]	-
26875	+	28.04.98	1.25	f	a.	a.[1]	positive[3]
27020	+	20.07.98	3	f	a.	-	negative[3]
34514	+	12.07.02	1.5	f	-	-	-
36716	+	13.01.04	2	f	a.	a.[1]	-
37514	+	09.09.04	2	f	a.	-	positive
42268	+	20.03.06	6	f	a.	-	negative[3]
42799	+	22.01.07	n.a.	f	a.	a.[1]	positive[1,3]
43484	+	02.04.08	2	f	a.	a.[2]	positive[2]
43660	+	06.04.09	5	f	a.	a.[1]	-
45019	+	15.09.10	2	f	-	-	-
45664	+	28.03.12	1.5	f	a.	a.[3]	positive[3]
49852	+	28.01.14	n.a.	n.a.	a.	-	positive
50773	+	29.12.14	3.5	f	a.	-	positive[2]
50898	+	07.12.15	n.a.	f	a.	-	positive
S11-0277	+	22.02.11	8	f	-	-	-
S11-0549	+	13.04.11	3	f	-	-	-
S14-1479	+	03.12.14	3	f	-	-	-

[1] Bouzalas et al. [1]; [2] Wuthrich et al. [2]; [3] Bouzalas et al. [3]; (a./–) = available/ not available; (m/f) = male/female.

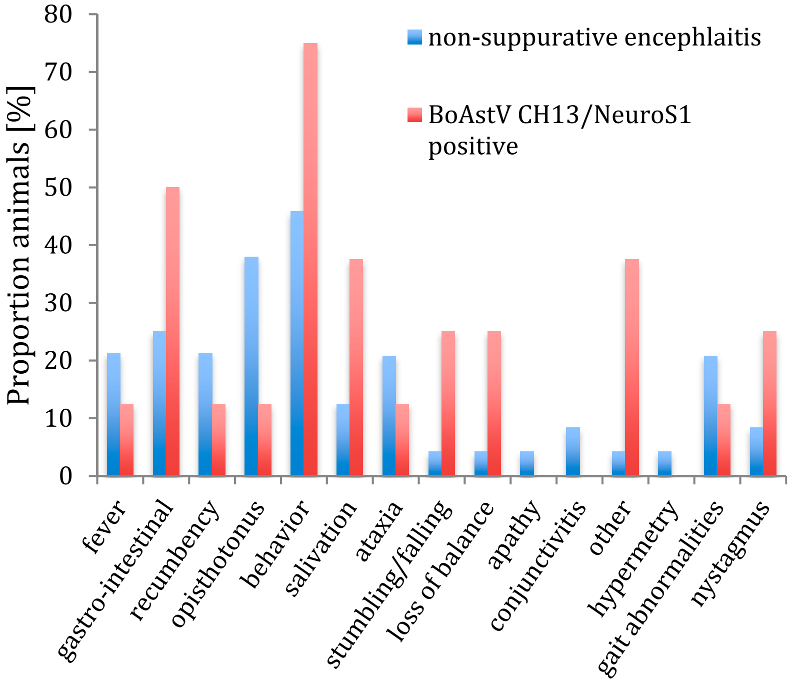

Figure S1. Anamnestic clinical information on non-suppurative encephalitis cases and BoAstV Ch13/NeuroS1-positive cases.

References

1. Bouzalas, I.G.; Wüthrich, D.; Selimovic-Hamza, S.; Drogemuller, C.; Bruggmann, R.; Seuberlich, T. Full-genome based molecular characterization of encephalitis-associated bovine astroviruses. *Infect. Genet. Evol.* **2016**, *44*, 162–168.
2. Wüthrich, D.; Boujon, C.L.; Truchet, L.; Selimovic-Hamza, S.; Oevermann, A.; Bouzalas, I.G.; Bruggmann, R.; Seuberlich, T. Exploring the virome of cattle with non-suppurative encephalitis of unknown etiology by metagenomics. *Virology* **2016**, *493*, 22–30.
3. Bouzalas, I.G.; Wüthrich, D.; Walland, J.; Drogemuller, C.; Zurbriggen, A.; Vandevelde, M.; Oevermann, A.; Bruggmann, R.; Seuberlich, T. Neurotropic astrovirus in cattle with nonsuppurative encephalitis in Europe. *J. Clin. Microbiol.* **2014**, *52*, 3318–3324.

© 2017 by the authors. Submitted for possible open access publication under the terms and conditions of the Creative Commons Attribution (CC-BY) license (http://creativecommons.org/licenses/by/4.0/).
